# Ocular Oncology in Brazil: What is our past, present, and future?

**DOI:** 10.5935/0004-2749.20210054

**Published:** 2025-02-02

**Authors:** Eduardo F. Marback, Virginia Laura Lucas Torres, Priscilla Lupi Ballalai Bordon, Marcelo Krieger Maestri, Luiz Fernando Teixeira

**Affiliations:** 1 Universidade Federal da Bahia, Salvador, BA, Brasil; 2 Sociedade Brasileira de Oncologia em Oftalmologia, São Paulo, SP, Brasil; 3 Universidade Federal de Pernambuco, Recife, PE, Brasil; 4 Universidade Federal do Rio Grande do Sul, Porto Alegre, RS, Brasil; 5 Departamento de Oftalmologia, Escola Paulista de Medicina, Universidade Federal de São Paulo, São Paulo, SP, Brasil

Ophthalmic oncology encompasses the care of tumors of the eye and ocular adnexa. In Brazil, ophthalmic oncology is not listed as a medical specialty by the Federal Council of Medicine, so it is a part of Ophthalmology^(1)^. In 1998, during the XIII Congresso Brasileiro de Prevenção da Cegueira e Reabilitação Visual in Rio de Janeiro, RJ, Brazil, a group of Ophthalmologists founded the *Sociedade Brasileira de Oncologia em Oftalmologia* (Brazilian Society of Ocular Oncology, SBOO). This group was led by Dr Clelia Maria Erwenne in collaboration with other great names in Brazilian Ophthalmology, including Drs Jacobo Melamed, Joaquim Marinho de Queiroz, José Vital Filho, José Wilson Cursino, Mário Motta, and Roberto L Marback, as well as a number of other younger physicians interested in the topic. Most of these great ophthalmologists were, at first, involved in the care of eye and adnexal tumors as a consequence of their primary area of interest in ophthalmology, such as pathology, pediatrics, genetics, the retina, orbital and eyelid surgery, uveal diseases, and cornea and external diseases^(2)^.

Looking back, the history of ophthalmic oncology, in Brazil and elsewhere, was no different from that of clinical oncology, surgical oncology, and other subareas of oncology. Here in Brazil, the Clinical and Surgical Oncology Societies were founded in the 1980s; before that, their activities were performed by clinicians and surgeons with special interest and dedication to the field. Similar to what occurred for clinical and surgical oncology, the pioneers of ophthalmic oncology were general ophthalmologists or ophthalmologists with training in other subareas who because of personal or circumstantial interest started to take care of eye cancer patients; a famous example was Prof. Sérgio Cunha from Universidade de São Paulo, a retinal surgeon who was renowned in laser treatment of intraocular tumors.

The contribution of Brazilian ophthalmologists to the care of eye cancer patients can be traced back many years. Hilário de Gouvêa first described the familial tendency in retinoblastoma in 1886^(3)^. In the 1950s and 1960s, Prof. Heitor Marback, interested in gaining a deeper knowledge of eye diseases, started to send enucleated eyes for pathologic study to the Armed Forces Institute of Pathology (AFIP; Washington, DC, USA), which was at that time headed by Dr Lorenz Zimmerman, who spread a long list of great ocular pathologists all over the world. This approach was amplified by Prof. Hilton Rocha, who started to encourage and send young Brazilian ophthalmologists and general pathologists to the specific ocular pathology training at the AFIP.

The first specific ambulatory care clinic for ocular cancer patients in Brazil was at the Hospital AC Camargo in the 1950s with Dr José Carlos Gouvea Pacheco. Dr Pacheco was the starting point of ophthalmic oncology, and Dr Clelia Maria Erwenne, whose primary initial focus was ophthalmic genetics, started to work with him during the 1980s. Dr Erwenne was the driving force of ophthalmic oncology in Brazil, training a great number of new ophthalmologists in the field of oncology and also providing an exemplary link for further specific training in many international services. In the next decade, she was involved with another eye cancer ambulatory care clinic in Escola Paulista de Medicina-Universidade Federal de São Paulo. This service worked in conjunction with the one in Hospital do Câncer AC Camargo up to 2000, at which time Dr Erwenne left the former.

Nowadays, oncology-trained ophthalmologists have spread to most of the Brazilian states and SBOO is present at all major Brazilian ophthalmic events. SBOO actively participates in general ophthalmic education programs as well as the Brazilian Ophthalmic Book Series. Another important achievement was the acceptance of ocular oncology and pathology, in a shared book with ocular plastic and orbital surgery, as the official theme for the 65^th^ Congresso Brasileiro de Oftalmologia (Brazilian Congress of Ophthalmology) to be held in Natal, RN in 2021. This publication is expected to function as a general reference in the ocular oncology field for Brazilian ophthalmologists.

At the present time, ocular oncology care is distributed over a series of universities and cancer hospitals throughout the country; unfortunately, some expensive treatment modalities, like plaque brachytherapy, are still a major problem for the public health system in every Brazilian state. We know that Brazil is a country of continental proportions with a number of social problems and many deficits in the public health system. These difficulties obviously affect ocular oncology services and patients. But we are also aware of the commitment of Brazilian ocular oncologists. Although still few in number, like most health professionals that choose to treat cancer patients, ocular oncologists are passionate people that do their very best to improve eye and adnexal cancer care in Brazil.
